# Impact of tRNA Modifications and tRNA-Modifying Enzymes on Proteostasis and Human Disease

**DOI:** 10.3390/ijms19123738

**Published:** 2018-11-24

**Authors:** Marisa Pereira, Stephany Francisco, Ana Sofia Varanda, Mafalda Santos, Manuel A. S. Santos, Ana Raquel Soares

**Affiliations:** Department of Medical Sciences, Institute of Biomedicine (iBiMED), University of Aveiro, 3810 Aveiro, Portugal; marisa.pereira@ua.pt (M.P.); s.marquesfrancisco@ua.pt (S.F.); sofiavaranda@ua.pt (A.S.V.); santos.mafalda@ua.pt (M.S.); msantos@ua.pt (M.A.S.S.)

**Keywords:** transfer RNA, tRNA modifications, tRNA-modifying enzymes, proteostasis, conformational disorders, translation, protein aggregation

## Abstract

Transfer RNAs (tRNAs) are key players of protein synthesis, as they decode the genetic information organized in mRNA codons, translating them into the code of 20 amino acids. To be fully active, tRNAs undergo extensive post-transcriptional modifications, catalyzed by different tRNA-modifying enzymes. Lack of these modifications increases the level of missense errors and affects codon decoding rate, contributing to protein aggregation with deleterious consequences to the cell. Recent works show that tRNA hypomodification and tRNA-modifying-enzyme deregulation occur in several diseases where proteostasis is affected, namely, neurodegenerative and metabolic diseases. In this review, we discuss the recent findings that correlate aberrant tRNA modification with proteostasis imbalances, in particular in neurological and metabolic disorders, and highlight the association between tRNAs, their modifying enzymes, translational decoding, and disease onset.

## 1. Introduction

Transfer RNAs (tRNAs) are the main players of translation machinery, carrying the amino acids required for nascent peptides being formed at the ribosome [1]. In humans, there are 610 genes that code for cytosolic and mitochondrial tRNAs (mt-tRNAs). These adaptor molecules recognize mRNA codons through their anticodons and decode the 20 standard amino acids of the cellular proteome, linking the genetic code information to amino acid identity [2]. Codon–anticodon recognition between the first and second bases of codons and the third and second bases of anticodons is determined by Watson–Crick pairing rules (A:U; U:A; G:C; C:G). On the other hand, the interaction between the third base of codons and the first base of anticodons (position 34 of the tRNA, also known as the wobble position) is more flexible, as it allows non-Watson–Crick base pairing (wobble hypothesis) [1,3]. This means that multiple codons can code for a single amino acid and a given tRNA may read more than one synonymous codon.

To ensure tRNA stability, translational efficiency, and fidelity, tRNAs are extensively modified post-transcriptionally like other RNA molecules, such as rRNA and mRNA [4]. To date, more than 80 tRNA modifications have been reported and an average of 13 modified bases can be found per tRNA molecule [5,6]. All of these modifications are catalyzed by different classes of tRNA-modifying enzymes and both cytosolic and mt-tRNAs are modified. Although several modifications are shared by both tRNA types, as is the case of N6-isopentenyl modification to adenosine (i6A) modification at position 37 of tRNAs [7], specific modifications are unique to a tRNA type, as is the case of taurine-derived modifications that are exclusively of mt-tRNAs [8].

Modifications occurring at or near the anticodon loop, in particular at positions 34 and 37, are highly conserved in eukaryotes and affect specific codon–anticodon interactions, regulating translational efficiency and fidelity. In fact, the vast majority of tRNA modifications occur at the wobble position (position 34), ensuring the correct codon–anticodon base pairing and reading frame maintenance while preventing translational frameshifting [9]. Modifications at this position are generally associated with decoding by increasing the diversity of codon recognition through codon–anticodon wobbling. Post-transcriptional modifications at base 37, adjacent to the anticodon loop, help to stabilize codon–anticodon interactions by improving intrastrand stacking interactions between codon and anticodon bases and within the anticodon loop [10].

**Figure 1:**
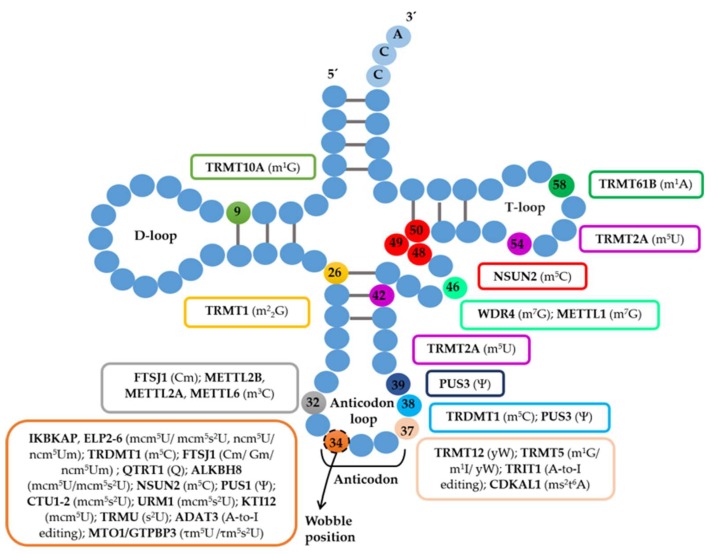
Schematic representation of the tRNA secondary structure with respective tRNA-modifying enzymes and modifications (in parenthesis). Connecting lines between RNA residues indicate base pairing. Abbreviations: tRNA, transfer RNA; m^1^G, 1-methylguanosine; m^2^_2_G, N2,N2-dimethyl guanosine; Cm, 2′-*O*-methylcytidine; m^3^C, 3-methylcytidine Gm, 2′-*O*-methylguanosine; ncm^5^Um, 5-carbamoylmethyl-2′-*O*-methyluridine; m^5^C, 5-methylcytosine; ncm^5^mU, 5-methoxycarbonylmethyluridine; mcm^5^s^2^U, 5-methoxycarbonylmethyl-2-thiouridine; Q, queuosine; s^2^U, 2-thiouridine τm^5^U, 5-taurinomethyluridine; τm^5^s^2^U, 5-taurinomethyl-2-thiouridine; I^6^A, N6-isopentenyladenosine; ms^2^t^6^A, 2-methylthio-N6-threonyl carbamoyladenosine; Ψ, pseudouridine; m^1^A, 1-methyladenosine.

Modifications occurring within the structural core of tRNAs, namely, in the D- or T- loops, are required for tRNA stability and functional folding [11], and their absence can lead to tRNA degradation and tRNA pool imbalances (Figure 1). The pseudouridine modifications, for example, stabilize specific structural motifs, and dihydrouridines help to maintain conformational flexibility (reviewed in [12]).

As previously mentioned, the wobble position is often modified in various tRNAs (Figure 1). In eukaryotes, 11 cytoplasmic tRNAs carry 5-methoxycarbonylmethyl (mcm^5^) or 5-carbamoymethyl (ncm^5^) modifications at Uridine 34 (U34). These modifications are catalyzed by the Elongator complex, a protein complex composed of six proteins, namely, ELP1 (or IKBKAP), ELP2, ELP3, ELP4, ELP5, and ELP6 [13]. ALKBH8 and its yeast homolog Trm9 are other methyltransferases required for the final step of mcm^5^ biogenesis at the wobble position [14,15]. Following mcm^5^U_34_ modification of tRNA^Lys(UUU)^, tRNA^Gln(UUG)^, and tRNA^Glu(UUC)^, a 2-thio group is added by ubiquitin-ligase-like proteins, namely, Urm1, Uba4, Ncs2, and Ncs6, resulting in 5-methoxycarbonylmethyl-2-thiouridine (mcm^5^s^2^) [16] (Figure 1). These modifications have been consistently correlated with translational fidelity and proteostasis [14,16,17,18,19,20,21].

U34 of a subset of mt-tRNAs contains taurine-derived modifications that are required for mitochondrial translation through codon–anticodon stabilization. Lack of this modification has been associated with MELAS and MERRF (myoclonus epilepsy with ragged red fibers), as fibroblast cells derived from MELAS and MERRF patients carrying A3243G mutations in mt-tRNA^Leu(UUR)^ and A8344G mutations in mt-tRNA^Lys^, respectively, lack the characteristic τm^5^U and τm^5^s^2^U modifications [22]. U34 of the human mt-tRNA^Lys^, mt-tRNA^Glu^, and mt-tRNA^Gln^ carry a m^5^s^2^ modification catalyzed by TRMU [23]. A particular mutation in this enzyme, namely, A10S, has been correlated with the phenotypic manifestations of deafness and results in mt-tRNA hypomodification, impairment of mitochondrial translation, and an increased level of reactive oxygen species [23,24].

Other nucleotides at and near the anticodon are also subjected to modifications (Figure 1). The Cytosine 34 (C34) and Cytosine 48 (C48) of tRNA^Leu(CAA)^ are methylated to 5-methylcytosine (m^5^C) by NSUN2 and the absence of these modifications leads to the accumulation of tRNA-derived fragments [25,26]. Guanosine 34 (G34) and cytidine 32 (C32) are methylated by the 2′-*O*-ribose-methyltransferase FTSJ1 in tRNA^Phe^ and lack of these modifications has been consistently correlated to X-linked mental retardation [27,28]. G34 in tRNAs with GUN anticodons, namely, tRNA^Asp^, tRNA^His^, and tRNA^Tyr^, is exchanged by queusine (Q) by the tRNA-ribosyltransferase (TGT) [29] (Figure 1). This modification is dependent on the availability of the substrate Q that in eukaryotes can only be retrieved by diet, as it is only synthesized de novo by bacteria. Recent studies have linked Q availability and modification to translation fidelity and genome recoding [30]. 

Adenosine (A) to inosine (I) (A-to-I) editing also occurs in tRNAs. The I modification is found at the wobble position as well as at positions 37 and 57. This modification is catalyzed by adenosine deaminases (ADATs) and expands the tRNA decoding capacity in ANN tRNAs as inosine-modified tRNAs can pair with U-, C-, and A-ended codons [31] (Figure 1). However, it is worth noting that I37 and I57 are further methylated to m^1^I37 or m^1^I57 and that m^1^I37 is only found in the eukaryotic tRNA^Ala^ [32].

N6-threonylcarbamoyladenosine (t6A) and i6A are conserved modifications found at A37 of several tRNAs. As other modifications at position 37, they are important to maintain the reading frame during decoding, promoting translation efficiency. t6A37 modifications occur in 5 mt-tRNAs, namely, tRNA^Ser^, tRNA^Thr^, tRNA^Asn^, tRNA^Ile^, and tRNA^Lys^, and low levels of this modification have been recently associated with MERFF [33], neurodegeneration [34], and diabetes [35]. i6A37 modification increases translation fidelity and efficiency of cognate codons, and i6A37 hypomodification is associated with mitochondrial defects in different organisms [7,36].

Methylation at G37 of tRNA^Phe^ is necessary for the formation of wybutosine (yW), which is crucial for the maintenance of the reading frame, preventing ribosome slippage on the phenylalanine UUU and UUC codons [10] (Figure 1). Uridines are often modified to pseudouridines at positions 38 and 39, broadening the decoding capacity of tRNAs (Figure 1). For example, modified tRNA^Leu(CAA)^ can decode stop UAG codons leading to stop-codon readthrough. In the absence of pseudouridine modification, the decoding ability of the tRNA is altered and it is not able to recognize the stop codon [37].

tRNA modifications have been extensively characterized in *Saccharomyces cerevisiae* and different studies correlate tRNA hypomodification with decreased translation accuracy and proteome imbalances in yeast [14,16,17,18]. Recent developments in next-generation sequencing [38], mass spectrometry [39], and ribosome profiling [40] have enabled the assessment and quantification of tRNA modifications and amino acid misincorporation [41,42], as well as its correlation with translation efficiency. This has contributed to significant advances in the field in the last years. In fact, the relevance of tRNA modifications in higher eukaryotes is starting to emerge, as deregulation of both tRNA modifications and tRNA-modifying enzymes has been found in several diseases, namely, neurological diseases, cancer, and mitochondrial-linked disorders. In this review, we focus on the recent findings that correlate aberrant cytosolic and mitochondrial tRNA modifications with proteostasis imbalances, with a particular focus on human conformational disorders. We highlight the association between tRNAs, their modifying enzymes and translation fidelity, and explore their therapeutic potential.

## 2. Proteome Disruption in Yeast upon U34 Hypomodification 

A growing number of reports show that tRNA hypomodification, in particular at the wobble position (position 34) or adjacent to the anticodon (e.g., position 37), affects translational accuracy and fidelity, leading to the accumulation of misfolded and aggregated proteins as well as activation of the cellular stress response. This is due to the fact that modifications at or near the anticodon have a direct impact in codon–anticodon interactions, ensuring base pairing flexibility and reading frame maintenance.

**Figure 2:**
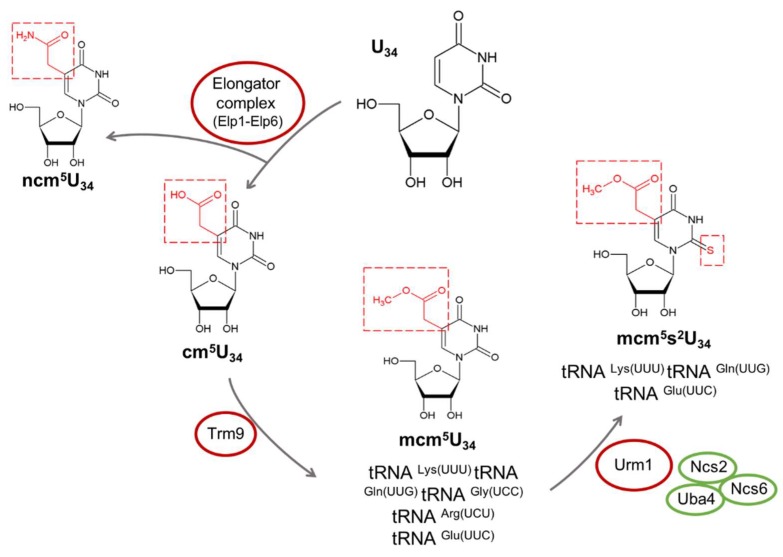
Yeast biosynthesis pathways of modified wobble uridines in different tRNA substrates catalyzed by the Elongator complex (Elp1–Elp6), Trm9, and Urm1 enzymes and the ubiquitin-ligase-like proteins, namely, Uba4, Ncs2, and Ncs6. In yeast, the Elongator complex (Elp1–Elp6) catalyze the wobble uridine (U_34_) modifications that form 5-carbamoylmethyluridine (ncm^5^U_34_) and 5-carboxymethyluridine (cm^5^U_34_). Then, the methyltransferase Trm9 uses cm^5^U_34_ as a substrate in different tRNAs: tRNA ^Lys(UUU)^ tRNA ^Gln(UUG)^, tRNA ^Gly(UCC)^, tRNA ^Arg(UCU)^, and tRNA ^Glu(UUC)^. Subsequent addition of a 2-thiol group by an enzyme cascade involving Urm1 and Uba4, Ncs2, and Ncs6 occurs in three of these tRNAs: (tRNA^Lys(UUU)^, tRNA^Gln(UUG)^, and tRNA^Glu(UUC)^). All modified nucleosides presented in this figure can be found in the MODOMICS database. Red dashed boxes represent the modification catalyzed by the respective enzymes in each step. Abbreviations: tRNA, transfer RNA; ncm^5^U, 5-carbamoylmethyluridine; cm^5^U, 5-carboxymethyluridine; mcm^5^U, 5-methoxycarbonylmethyluridine; mcm^5^s^2^U, 5-methoxycarbonylmethyl-2-thiouridine.

U34 mcm^5^ and ncm^5^ modifications are catalyzed by the Elongator complex [13], which is also engaged in transcriptional elongation and histone acetylation [43]. In yeast, this complex catalyzes the formation of cm^5^U_34_ that is then used as a substrate by the methyltransferase Trm9 for the formation of mcm^5^U_34_ of tRNA^Lys(UUU)^, tRNA^Gln(UUG)^, tRNA^Gly(UCC)^, tRNA^Arg(UCU)^, and tRNA^Glu(UUC)^. Three of these tRNAs, namely, tRNA^Lys(UUU)^, tRNA^Gln(UUG)^ and tRNA^Glu(UUC)^, are further modified at the wobble position with a 2-thio group, resulting in mcm^5^s^2^ nucleotide, catalyzed by Urm1 and other ubiquitin-ligase-like proteins, namely, Uba4, Ncs2 and Ncs6 [9,14,16] (Figure 2). Disruption of these modifications leads to proteome instability in yeast. For example, Trm9 yeast mutants are deficient in 2 of 23 tRNA modifications, namely, mcm^5^U and mcm^5^s^2^U, as determined by LC-MS. The absence of these modifications in Trm9 mutants leads to amino acid misincorporations and frameshifting errors during the translation of specific codons, namely, those belonging to arginine, glutamic acid, glutamine, and lysine mixed codon boxes, linking these wobble base modifications to translational fidelity [44]. Increase in translational errors in Trm9 mutants is accompanied by activation of the unfolded protein response (UPR) and activation of the heat shock response, key protein quality control mechanisms that are activated to refold or clear unfolded proteins, preventing the accumulation of toxic protein aggregates in cells [44]. Moreover, translation of AGA and GAA codons is enhanced in the presence of the modifications catalyzed by Trm9 as well as the translation elongation speed through these codons relative to Trm9 yeast mutants [45]. The absence of Trm9 results in hypomodification of tRNA^Arg(UCU)^ and tRNA^Glu(UUC)^ and is directly correlated with a decrease in the synthesis of proteins enriched in AGA and GAA codons, mainly due to ribosome stalling during translation. The vast majority of these proteins are involved in protein synthesis, cell cycle control, and DNA damage response and are consistently downregulated either under normal or stress conditions in Trm9 mutants, showing that lack of modifications impairs the cell’s ability to respond to stress [14]. Taken together, these results suggest that tRNA modifications are important for the regulation of codon-biased translation.

Similarly, yeast cells lacking Elp3, one of the components of the Elongator complex, and Urm1, which together are required to generate the mcm^5^s^2^U modification at U34 of tRNA^Lys^, tRNA^Glu^, and tRNA^Gln^, have lower resistance to stress due to inefficient translation of stress-related genes enriched for AAA, CAA, and GAA codons [16,46]. Interestingly, overexpression of tRNA^Lys(UUU)^ is sufficient to reverse the stress defects of *Schizosaccharomyces Pombe* Elp3 mutant [46]. Another double yeast mutant for Elp3 and Uba4 that also lacks mcm^5^s^2^U displays impaired protein synthesis that is partially restored by overexpression of hypomodified tRNA^Lys(UUU)^ [47].

Also, overexpression of hypomodified tRNA^Lys(UUU)^, tRNA^Glu(UUC)^, and tRNA^Gln(UUG)^ restore CAA and AAA codon translation rates and protein homeostasis in Ncs2 and Elp6 yeast mutants that lack 2-thiolation and mcm^5^/ncm^5^ modifications, respectively [18]. Ribosome profiling of these mutants revealed increased ribosome occupancy at CAA and AAA codons, indicative of a translational slowdown. Moreover, proteotoxic stress is triggered in these yeast mutants, probably as a response to the accumulation of protein aggregates upon aberrant U34 modification. Metastable proteins are the class of proteins with the highest tendency for aggregation upon loss of U34 modifications, and Ncs2Elp6 double mutants have impaired ability to restore proteostasis [18]. However, contrary to the study by Deng et al. [14], Ncs2Elp6 double yeast mutants lacking U34 mcm^5^s^2^ modifications do not display an impaired translation of stress response genes enriched in codons affected by U34 hypomodification but rather a chronic protein misfolding that causes a reduced ability for cells to restore proteostasis [18]. Since both studies used different yeast strains and mutants, it is possible that these divergent results may translate differences in genetic backgrounds. 

Accumulation of protein aggregates is also observed in budding yeast lacking U34 mcm^5^s^2^ and pseudouridine (ψ) at positions 38 in tRNA^Gln(UUG)^ [17]. These mutants also display an impaired synthesis of the Gln-rich prion Rnq1. Similar to other studies [18,46], translational defects are rescued upon tRNA^Gln(UUG)^ overexpression, indicating that both mcm^5^s^2^U and ψ38 are key for this tRNA decoding [17]. 

Taken together, these studies demonstrate that deregulation of tRNA modifications mainly affects protein synthesis rate. In fact, absence of wobble modifications leads to ribosome stalling in particular codons and expression deregulation of protein subsets enriched in those codons. Altered levels of these proteins may trigger the cellular stress response or affect the cell′s ability to respond to stress conditions. Besides aberrant protein synthesis, tRNA hypomodification, in particular of U34, is directly correlated with accumulation of protein aggregates and proteostasis imbalances that may result from ribosome stalling and subsequent protein folding defects. As overexpression of hypomodified tRNAs restores both translation rates and proteostasis, further studies are needed to elucidate what is the underlying cause of the accumulation of misfolded proteins. 

Since most of these tRNA modifications, as well as the respective tRNA-modifying enzymes, are conserved among eukaryotes, it is reasonable to speculate that similar phenotypes can occur in higher eukaryotes and that tRNA hypomodification can be an underlying cause of several human diseases where proteostasis is affected. One of the first studies recapitulating similar phenotypes in higher eukaryotes was performed in *Caenorhabditis elegans*. A U34 2-thiolation-deficient *C. elegans* strain displayed ribosome stalling at AAA, CAA, and GAA codons, similar to the yeast Ncs2 mutant [18]. These mutant animals also had a 2.5-fold higher protein aggregate burden when compared to the controls as well as an increase in the expression of heat shock proteins, indicating that tRNA hypomodification at U34 disrupts proteostasis in nematodes [18].

Proteostasis imbalances together with deregulation of the UPR are often observed in Alzheimer′s, Parkinson′s, or amyotrophic lateral sclerosis (ALS), as well as in type 2 diabetes and cancer [48,49,50]. Several tRNA-modifying enzymes were previously found to be deregulated or mutated in most of these disorders, but only recently have researchers started focusing on the correlation between tRNA modifications and the translation imbalances observed in these disorders. In the next section, the recent advances establishing a link between human diseases and tRNA modification deregulation are discussed.

## 3. Deregulation of tRNA Modifications in Protein Conformational Diseases

The first report of a tRNA mutation linked to disease was a point mutation in the mitochondrial tRNA^Leu(UUR)^ gene in MELAS (mitochondrial myopathy, encephalopathy, lactic acidosis, and stroke-like episodes) discovered in 1990 [51]. Since then, several tRNA mutations, as well as deregulation and/or mutation of tRNA-modifying enzymes, have been reported in a panoply of human diseases ranging from neurological disorders to metabolic imbalances and cancer [52]. 

From the analysis of the existing literature, it is possible to infer that while tRNA modifications are generally negatively affected in metabolic and neurodegenerative diseases [20,27,53,54,55,56], hypermodified tRNAs and upregulation of tRNA-modifying enzymes are often found in different cancers [57,58]. It is worth noting that the same tRNA-modifying enzyme can be affected differently in unrelated diseases, ultimately affecting the levels of tRNA modifications catalyzed by it. For example, mutations in NSUN2, a tRNA-modifying enzyme that methylates cysteine to m^5^C, are associated with neurological defects and tRNA hypomodification [59,60], while upregulation of this enzyme has been found in different tumors and has been associated with poor prognosis of head and cancer squamous carcinoma [61,62].

It is possible that these observations are correlated with the different types of proteome imbalances observed in these disorders. While neurodegenerative disorders are characterized by the generation of unfolded proteins that accumulate as protein aggregates [48,63], protein synthesis rate is generally upregulated in cancer. Indeed, tumors are characterized by increased cellular proliferation and increased protein synthesis rate, but not all tRNAs are equally upregulated in cancer, raising the possibility that cancer cells have distinct tRNA pools that may more efficiently translate subsets of oncogenes [57,58]. For example, upregulation of the initiator tRNA^Met^ [64] as well as upregulation of tRNA-modifying enzymes [61,65] contribute to cancer initiation and progression. 

### 3.1. Role of Elongator Complex, mcm^5^, ncm^5^, and mcm^5^s^2^ Modifications in Neurological Disorders

**Figure 3:**
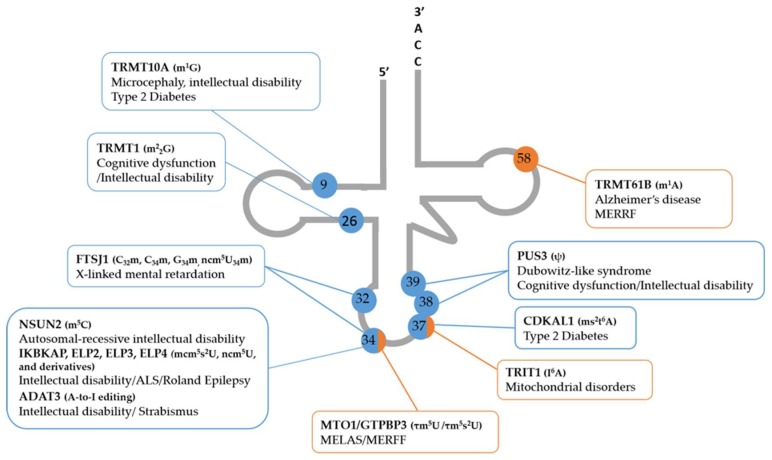
Identification of tRNA-modifying enzymes and respective tRNA modification sites involved in neurological and metabolic disorders. Schematic representation of the clover leaf tRNA secondary structure including tRNA-modifying enzymes (in bold), modifications (in parenthesis), and human diseases associated with cytosolic and mitochondrial tRNA defects marked in blue and orange, respectively. Abbreviations: tRNA, transfer RNA; m^1^G, 1-methylguanosine; m^2^_2_G, N2,N2-dimethyl guanosine; Cm, 2′-*O*-methylcytidine; Gm, 2′-*O*-methylguanosine; ncm^5^Um, 5-carbamoylmethyl-2′-*O*-methyluridine; m^5^C, 5-methylcytosine; ncm^5^mU, 5-methoxycarbonylmethyluridine; mcm^5^s^2^U, 5-methoxycarbonylmethyl-2-thiouridine, τm^5^U, 5-taurinomethyluridine; τm^5^s^2^U, 5-taurinomethyl-2-thiouridine; I^6^A, N6-isopentenyladenosine; ms^2^t^6^A, 2-methylthio-N6-threonyl carbamoyladenosine; Ψ, pseudouridine; m^1^A, 1-methyladenosine; ALS, amyotrophic lateral sclerosis; MELAS, mitochondrial encephalomyopathy, lactic acidosis, and stroke-like episodes; MERFF, myoclonus epilepsy associated with ragged red fibers.

Mutations in the donor splice site of intron 20 of *ELP1/IKBKAP* gene (an Elongator complex subunit) have been linked to familial dysautonomia (FD) (Figure 3; Table 1), a hereditary genetic disorder characterized by improper development and function of the sensorial and autonomic nerve systems [66]. In fact, most cases of FD (99.5%) result from a single nucleotide point mutation in the *IKBKAP* gene [67], and brain tissue as well as fibroblast cell lines from FD patients have reduced levels of mcm^5^s^2^U modification [68]. Hypomodification of the wobble uridine of tRNAs for Val, Gly, Thr, and Arg of FD patient cells was reverted after rectifying the aberrant splicing of *IKBKAP* pre-mRNAs harboring an FD-causing mutation [69]. Recovery of tRNA modification levels led to the concomitant recovery of cell growth [69]. Consistently, *elp1 C. elegans* mutants that lack mcm^5^s^2^U nucleosides display impaired protein synthesis and deficient chemosensory neurons [70]. Moreover, knockout of *Ikbkap* results in embryonic lethality in mice, while mice central nervous system conditional knockouts of this protein are characterized by developmental neurological defects [71]. Also, *Ikbkap* conditional knockouts in mice testes display defects in meiotic progression and significantly reduced levels of mcm^5^U, ncm^5^U, and mcm^5^s^2^U [72]. Conditional *Ikbkap*/*Elp1* knockout mice recapitulate FD hallmarks and also display reduced levels of mcm^5^s^2^U nucleosides [19]. Moreover, translation of large, AA-biased genes involved in DNA damage repair, such as *Brca2*, was negatively affected in these knockouts, resulting in increased DNA damage and UPR activation [19], similar to what was previously observed in yeast [18]. Taken together, these results establish an association between Elongator complex dysfunction, defects in tRNA modification, inefficient translation, UPR, and FD onset and progression.

ELP3 has also been associated with neurological defects (Figure 3; Table 1). Allelic variants of the *ELP3* gene have been associated with sporadic ALS, a neurodegenerative disease characterized by degeneration of motor neurons [53]. Mutations in *SOD1*, *FUS*, *C9orf72*, and *TDP*-*43* are also related to ALS onset, and wildtype ELP3 expression is associated with increased survival of patients carrying a *C9orf72* repeat expansion [73]. This tRNA-modifying enzyme is also required for proper neuronal function and survival, as drosophila and zebrafish embryos lacking ELP3 develop motor axonal abnormalities that recapitulate the phenotypes induced by mutant SOD1 and TDP-43 [53]. Moreover, axonopathy was attenuated after ELP3 coexpression in two ALS zebrafish models, namely, SOD1 and C9Orf72 mutants, reinforcing the protective role of ELP3 in ALS [20]. On the other hand, deletion of ELP3 in mice is lethal and constitutive heterozygous deletion of ELP3 accelerates disease onset and leads to a decrease of mcm^5^s^2^U nucleosides in tRNAs [20]. Ribosome profiling of the forebrains of another ELP3 conditional knockout mouse model confirmed ribosome stalling of codons read by ncm^5^/mcm^5^-modified tRNAs and UPR activation [74]. Similarly, ELP3 *C. elegans* mutants lack ncm^5^U and mcm^5^s^2^U tRNA modifications and exhibit neurological and developmental defects as well as reduced translation [70]. Moreover, silencing of ELP3 in NSC34 cells leads to a 28% increase in total protein aggregation and increases the level of insoluble mutant human SOD1. ELP3 overexpression reduces the amount of insoluble SOD and restores the levels of mcm^5^s^2^U in approximately 50%, correlating this modification with SOD1 solubility [20]. Importantly, the reduced levels of ELP3 are directly correlated with the levels of mcm^5^s^2^U in motor cortices of ALS sporadic patients, suggesting that ELP3 is a modifier of the disease by affecting protein aggregation of particular proteins through the control of the wobble modification [20,74].

Mutations in other Elongator complex subunits, namely, *ELP2* and *ELP4*, have been correlated with neurodevelopmental disabilities and rolandic epilepsy, respectively (Figure 3; Table 1). *ELP2* mutations were identified by next-generation sequencing in intellectual disability patients [54,75]. *ELP2* Single-nucleotide polymorphisms (SNPs) were also identified by genome-wide association studies (GWAS) in patients with frontotemporal dementia [76]. The association of *ELP4* with rolandic epilepsy is contradictory, as there are studies identifying this gene as a risk locus for the disease [77] and studies demonstrating that there is no association of *ELP4* SNPs with centrotemporal spikes [78]. However, to date, there are no studies correlating *ELP2* or *ELP4* mutations with translational alterations and deregulated levels of tRNA wobble modifications in these disorders, but given the consequences of ELP1 and ELP3 deregulation in other neurological disorders and that neurons are extremely sensitive to increased translational error rate [37], it is expected that tRNA hypomodification and translation slow-down also occur in the absence of other Elongator complex components.

### 3.2. Impact of Other Anticodon Modifications in Neurological and Metabolic Diseases 

Besides uridylations and thiolations, the wobble position of tRNAs is subjected to other modifications such as m^5^C, Gm34, and A-to-I editing catalyzed by NSUN2, FTSJ1, and ADAT3, respectively. FTSJ1 also catalyzes Cm32 modifications and NSUN2 also methylates C47 and C48. 

FTSJ1 mutations are linked to nonsyndromic X-linked intellectual disability (Figure 3; Table 1), a genetically and clinically heterogeneous group of brain disorders [27,28,79]. Patient cell lines bearing disease-causing FTSJ1 mutations display 2′-*O*-methylation hypomodification at the anticodon loop of tRNA^Phe^, providing evidence that FTSJ1 also catalyzes this modification in humans and that lack of Gm34 may be a trigger for X-linked intellectual disability [27]. 

Different studies implicate mutations in NSUN2 in autosomal-recessive intellectual disability (Figure 3; Table 1), and drosophila mutants lacking the NSUN2 ortholog have severe short-term memory deficits, linking NSUN2 and RNA methylation to cognitive development [55,59]. A splicing mutation in NSUN2 is also present in Dubowitz-like syndrome patients that are characterized by microcephaly, mental retardation, and peculiar faces. This mutation is sufficient for reduction of both NSUN2 mRNA and protein levels in cultured fibroblast from patients and results in the loss of m^5^C modifications on C47 and C48 of the tRNA^Asp(GTC)^, a NSUN2 substrate [60]. NSUN2 mutant mice show reduced m^5^C at C34 of NSUN2 substrate tRNA^Leu(CAA)^ [93]. Moreover, mice lacking two methyltransferases, namely, NSUN2 and DNMT2, are also characterized by m^5^C loss in tRNAs followed by tRNA degradation, developmental defects, and lethality. These double mutants are also characterized by decreased protein synthesis rate [94], which is also observed in NSUN-2-deficient mouse brains [26], correlating once again tRNA modifications with protein synthesis regulation. DNMT2 (TRDMT1) polymorphisms have been also associated with spina bifida risk and increased folate levels in red blood cells [95].

A single missense mutation identified in ADAT3 by exome sequencing can cause severe intellectual disability and strabismus [87,88,96], implicating another gene involved in protein translation in the development of neurological diseases (Figure 3; Table 1). Recently, a second mutation in ADAT3 was reported in a patient with mild intellectual disability [97]. Although the levels of A-to-I editing in the patients carrying these mutations were not analyzed, the resulting phenotypes are similar to the ones obtained when other tRNA-modifying enzymes that catalyze wobble modifications are mutated. This reinforces the fact that wobble modifications, independently of which type, are crucial to translation efficiency and disturbance of these modifications affect the translation of essential genes for brain development. 

Deficiency in MTO1, the enzyme that catalyzes taurine modifications, leads to complete loss of this modification in mt-tRNAs in both cell lines and mice, as well as impaired mitochondrial translation, accumulation of misfolded mitochondrial proteins, and UPR activation [21]. Human fibroblasts carrying a homozygous mutation in the *MTO1* also display hypomodification of mt-tRNAs and upregulation of proteostasis stress-related genes [98]. Treatment of MTO1-deficient cells with the chemical chaperone TUDC, that has been tested successfully in ALS patients [99], alleviated the accumulation of protein aggregates and suppressed cytotoxic UPR [21], indicating that chemical chaperones may be considered as a therapeutic strategy to target proteotoxic stress. Pathogenic mutations in GTPBP3, another enzyme required for taurine modification, results in enzymatic activity loss and decreased levels of taurine modification in mt-tRNAs of a two-year-old patient with a mitochondrial disorder [8] (Figure 3; Table 1).

Disturbances of modifications at position 37 are particularly linked to metabolic dysfunction and proteostasis imbalances. The mammalian methylthiotransferase CDKAL1, essential for the generation of ms^2^t^6^A_37_ in cytoplasmic tRNA^Lys(UUU)^, is required for the accurate translation of AAA and AAG codons [89,100] (Figure 3; Table 1). Mice lacking CDKAL1 display a reduction in insulin β-cells secretion and endoplasmic reticulum (ER) stress caused by misreading of lysine codons in proinsulin. This indicates a link between deficient translation in the absence of CDKAL1 and increased risk of type 2 diabetes [35,90]. In drosophila, lack of N6-threonylcarbamoyadenosine (t^6^A) at position 37 of tRNAs leads to the accumulation of aberrant proteins in the lumen of the ER and activation of the UPR [101]. Recently, a t^6^A biosynthesis defect in humans due to a mutation in KAE1, a component of the KEOPS complex that catalyzes the second biosynthetic step of t^6^A, was reported [34]. This mutation is associated with renal tubulopathy and also with severe neurodegeneration [34]. Besides, mutant mt-tRNA^Thr^ bearing the A15923G mutation isolated from MERFF-like patient fibroblast and myoblasts displays low levels of t^6^A37, linking this hypomodification to pathological conditions [33].

TRIT1, a tRNA isopentenyl transferase that catalyzes i^6^A37 modification in mitochondrial and cytosolic tRNAs is mutated in patients with mitochondrial disorders (Figure 3; Table 1). This mutation causes i^6^A_37_ deficiency that is reversed by transfection of wildtype TRIT1 in patient′s fibroblasts [7]. 

### 3.3. tRNA-Modifying Enzymes that Catalyze tRNA Modifications outside the Anticodon Are Also Associated with Disease 

Mutations in the *TRMT1* gene, which encodes an enzyme that demethylates guanosines at position 26 of several tRNAs, have also been identified as the cause of certain forms of autosomal-recessive intellectual disability [56] (Figure 3; Table 1). A recent study demonstrated that TRMT1 catalyzes dimethylguanosine (m2,2G) modification in both mitochondrial and nucleus-encoded tRNAs, as TRMT1-deficient cells lacked these modifications [80]. Expression of TRMT1 variants found in intellectual disability patients in TRMT1 knockout cells is not sufficient to catalyze the m2,2G modification at G26, whereas transfection of wildtype TRMT1 is able to recover tRNA G26 modifications. Moreover, protein synthesis is negatively affected in TRMT1 knockout cells and redox homeostasis is impaired, particularly in neural stem cells, indicating that this enzyme is important to oxidative stress resistance and may regulate the translation of stress-response genes through m2,2G modification [80].

A homozygous truncation mutation in PUS3 is also associated with intellectual disability [86] (Figure 3; Table 1). Cells derived from patients bearing this mutation show decreased levels of isomerization of uracil in positions 38 and 39 of tRNA^Leu^, which require PUS3 activity, but no alterations in the levels of other modifications [86]. 

TRMT10A is a tRNA-methyltransferase that catalyzes methylation of guanine-9 (m^1^G_9_). Several studies have reported TRMT10A nonsense mutations in young-onset diabetes and microcephaly, which is correlated with its enriched expression in the pancreas and brain [81,82,83,84] (Figure 3; Table 1). Recently, tRNA^Gln^ and tRNA^iMet^ were identified as TRMT10A targets. m^1^G is significantly reduced in TRMT10A-deficient patient lymphoblasts and TRMT10A depletion induces β-cell oxidative stress and generation of tRNA-derived fragments [85].

Decreased expression in the methyltransferase TRMT61B, which catalyzes m^1^A_58_ in three mt-tRNA (mt-tRNA^Leu(UUR)^, mt-tRNA^Ser(UCN)^, and mt-tRNA^Lys^) [102], has been observed in Alzheimer′s disease [91] (Figure 3; Table 1). However, it is still unclear if the reduction of TRMT61B-mediated methylation, or rather hypomethylation, of a specific TRMT61B substrate is responsible for disease development. Nevertheless, lack of m^1^A_58_ modification is found in tRNA^Lys^ of patients carrying the mitochondrial DNA mutation m.8344A > G associated with MERRF and is correlated with decreased protein synthesis rate. Overexpression of TRMT61B in MERRF patient myoblasts is sufficient to restore m^1^A_58_ modification levels in tRNA^Lys^ and increase the synthesis of selected proteins [92], indicating that modifications of mt-tRNAs impact mitochondrial gene expression.

## 4. tRNA Hypomodification and Generation of tRNA-Derived Fragments

tRNA-derived fragments are a heterogeneous class of molecules that derive from mature tRNAs and play regulatory roles in a variety of cellular processes, namely, gene expression, translation initiation and elongation, and stress granule assembly, among others. Many tRNA-derived fragments have been implicated in cancer, neurodegenerative disorders, and infection, and emerging data suggest that tRNA modifications play a critical role in the generation and accumulation of tRNA fragments in human diseases [103]. In fact, tRNA hypomodification has been associated with the accumulation of particular tRNA-derived fragments. For example, deletion of NSUN2 in mice and humans induces the accumulation of 5´tRNA-derived fragments in brains as a result of m^5^C tRNA hypomodification [26,104]. The accumulation of tRNA derived fragments triggers cellular stress responses and, consequently, reduces protein translation rates, leading to decreased brain size and affected synapse development in NSUN2 knockout mice embryos [26]. Moreover, inhibition of angiogenin-mediated tRNA cleavage rescues the elevated stress levels of NSUN2(−/−) cells during neurodevelopment in vivo [26], suggesting the protective role of NSUN2 modification in human neurocognitive and intellectual disabilities. Taken together, these results show that m^5^C inhibit tRNA cleavage and that NSUN2 is required for brain development.

Loss of TRMT10A in both lymphoblasts and iPSC-derived β-like cells leads to tRNA m^1^G_9_ hypomethylation, tRNA^Gln^ cleavage, and generation of 22 nucleotides and longer 5′tRNA^Gln^ fragments [85]. These fragments are able to promote β-cell apoptosis, which is reversed by transfection of a specific 5′tRNA^Gln^ antisense oligonucleotide [85], suggesting a role of tRNA-derived fragments in the development of diabetes and β-cell dysfunction as a result of tRNA hypomodification.

Pseudouridylation of RNA catalyzed by PUS7 has been recently identified as a major activator of a network of tRNA-derived fragments involved in translation regulation in embryonic human stem cells (hESCs) [105]. Interestingly, PUS7 knockout in hESCs have increased de novo protein synthesis, and tRNA^Tyr(GUA)^ was identified as a PUS7 target. Moreover, loss of PUS7 leads to a decrease, rather than an increase, of particular 5′tRNA-derived fragments containing a 5′terminal oligoguanine (TOG), derived from tRNA^Ala^, tRNA^Cys^, and tRNA^Val^ that are also PUS7 modified. In fact, these pseudouridylated 5′ tRNA-derived fragments inhibit translation initiation, which is required for translation control for accurate stem cell fate determination [105].

Taken together, these data indicate that lack of tRNA modifications has a direct impact on the generation of tRNA-derived fragments implicated in pathological cellular processes. However, additional studies are required to fully understand the correlation between tRNA modifications and tRNA-derived fragments and how this is affected in different cell types and conditions.

## 5. Conclusions and Future Perspectives

Elucidation of the roles of tRNA modifications and tRNA-modifying enzymes in human disease is starting to emerge. Several genetic mutations in tRNA-modifying enzymes have been associated with pathological conditions, in particular, neurological and metabolic disorders, suggesting that hypomodification of tRNAs contribute to the onset and/or development of human diseases (Figure 3/Table 1). Recent advances in the role of the Elongator complex in the development of neurological disorders has been crucial to highlight the importance of tRNA modifications in proteostasis and human disease. It is also clear that although modifications at the wobble position are fundamental for proteostasis, modifications in other tRNA sites seem to be equally relevant. It would be interesting to perform large-scale screenings in mammalian cells to pinpoint which and how tRNA modifications are involved in translation accuracy, efficiency, protein misfolding, and aggregation. 

The emerging studies indicate that lack of particular modifications affects particular subsets of tRNAs, affecting the translation of specific codons and protein subsets. The recent advances in ribosome profiling, tRNA and mRNA sequencing, and mass spectrometry will undoubtedly contribute to elucidate how tRNA modifications affect translation and proteostasis in disease contexts and, equally important, which proteins are mainly affected by different tRNA processing defects.

The correlation between tRNA modifications and protein synthesis regulation suggests that RNA modifications participate in the epigenetic control of gene expression at the translational level, but further studies are necessary to clarify this issue. It is also important to keep in mind that some tRNA-modifying enzymes have alternative noncanonical functions beyond tRNA modifications, and that mutations in heterodimeric tRNA-modifying enzymes may directly affect other cellular processes where these enzymes are involved, contributing to the phenotypes observed in particular diseases. Additionally, the discovery of tRNA-derived fragments and their recent implications in many diseases will provide valuable insights into human pathogenic mechanisms and will better clarify the implications of tRNA modifications in the regulation of tRNA cleavage. It is still unclear whether certain human pathologies associated with tRNA modifications and tRNA-modifying enzymes arise as a direct consequence of altered translation of mRNAs or whether other uncharacterized mechanisms are involved, but it is expected that this will be a main focus of research in this field.

## Figures and Tables

**Table 1:** List of tRNA ModEnz, tRNA modifications, and tRNA deregulations associated with neurological and metabolic disorders.

tRNA ModEnz	tRNA Modifications	Neurological and Metabolic Disorders	tRNAs Deregulated	References
IKBKAP (ELP1)	mcm^5^U_34,_ mcm^5^s^2^U_34_, ncm^5^U_34_, ncm^5^Um_34_	Familial dysautonomia (FD)	tRNA^Gln (UUG)^ tRNA^Lys (UUU)^	[19,66,67,68,69]
ELP2		Neurodevelopmental disabilities	Several	[54,75,76]
ELP3		Amyotrophic lateral sclerosis (ALS)	tRNA^Gln (UUG)^ tRNA^Lys (UUU)^ tRNA^Glu (UUC)^	[20,53]
ELP4		Rolandic epilepsy	Several	[77,78]
FTSJ1	mC_32_, mC_34_, mG_34,_ ncm^5^mU_34_	Nonsyndromic X-linked intellectual disability	tRNA^Leu^ tRNA^Phe^ tRNA^Trp^	[27,28,79]
TRMT1	m^2,2^G_26_	Autosomal-recessive intellectual disability;	Several	[56,80]
NSUN2	m^5^C_34_, m^5^C_48_, m^5^C_49,_ m^5^C_50_	Autosomal-recessive intellectual disability; Dubowitz-like syndrome	tRNA^Leu (CAA)^ tRNA^Gly (GCC)^	[55,59,60]
TRMT10A (RG9MTD2)	m^1^G_9_	Microcephaly, epilepsy, intellectual disability, type 2 diabetes	Several	[81,82,83,84,85]
PUS3	ψU_38_, ψU_39_	Autosomal-recessive intellectual disability	tRNA^Phe^	[86]
ADAT3	A-to-I editing	Intellectual disability	tRNA^Ala, Pro, Thr^ tRNA^Val, Ser, Arg^ tRNA^Leu, Ile^	[87,88]
CDKAL1	ms^2^t^6^A_37_	Type 2 diabetes	tRNA^Lys (UUU)^	[35,89,90]
TRMT61B	m^1^A_58_	Alzheimer´s disease MERRF	mt-tRNA^Leu (UUR)^ mt-tRNA^Lys (UCN)^ mt-tRNA^Ser^	[91,92]
MTO1	τm^5^U_34_	MELAS	mt-tRNA^Leu(UUR)^	[8,21,22]
GTPBP3	τm^5^s^2^U_34_	MERRF	Mt-tRNA^Lys^	[8,22]
TRIT1	I^6^A_37_	Mitochondrial disorders	Mt-tRNA^Ser(UCN)^	[7]
